# An observational study identifying obese subgroups among older adults at increased risk of mobility disability: do perceptions of the neighborhood environment matter?

**DOI:** 10.1186/s12966-015-0322-1

**Published:** 2015-12-18

**Authors:** Abby C. King, Deborah Salvo, Jorge A. Banda, David K. Ahn, Thomas M. Gill, Michael Miller, Anne B. Newman, Roger A. Fielding, Carlos Siordia, Spencer Moore, Sara Folta, Bonnie Spring, Todd Manini, Marco Pahor, for the LIFE Study Investigators

**Affiliations:** Health Research & Policy, and Stanford Prevention Research Center, Department of Medicine, Stanford University School of Medicine, 259 Campus Drive, HRP Redwood Building, Room T221, Stanford, CA USA; Stanford Prevention Research Center, Department of Medicine, Stanford University School of Medicine, Stanford, CA USA; Michael and Susan Dell Center for Healthy Living, the University of Texas Health Science Center at Houston, School of Public Health (Austin Regional Campus), Austin, TX USA; Department of Medicine, Yale University School of Medicine, New Haven, CT 06504 USA; Department of Biostatistical Sciences, Lake Forest School of Medicine, Winston-Salem, NC 27157 USA; Healthy Aging Research Program, University of Pittsburgh, Pittsburgh, PA 15213 USA; Jean Mayer USDA Human Nutrition Research Center on Aging, Tufts University, Boston, MA 02111 USA; Health Promotion, Education, and Behavior, University of South Carolina, Columbia, SC 29208 USA; Department of Medicine, Northwestern University Feinberg School of Medicine, Chicago, IL USA; Department of Aging and Geriatric Research, University of Florida, Gainesville, FL 32608 USA

**Keywords:** Obesity, Aging, Perceived environment, Neighborhood, Mobility, Disability, Residential density, Race/ethnicity, Homophily, Recursive partitioning

## Abstract

**Background:**

Obesity is an increasingly prevalent condition among older adults, yet relatively little is known about how built environment variables may be associated with obesity in older age groups. This is particularly the case for more vulnerable older adults already showing functional limitations associated with subsequent disability.

**Methods:**

The Lifestyle Interventions and Independence for Elders (LIFE) trial dataset (*n* = 1600) was used to explore the associations between perceived built environment variables and baseline obesity levels. Age-stratified recursive partitioning methods were applied to identify distinct subgroups with varying obesity prevalence.

**Results:**

Among participants aged 70–78 years, four distinct subgroups, defined by combinations of perceived environment and race-ethnicity variables, were identified. The subgroups with the lowest obesity prevalence (45.5–59.4 %) consisted of participants who reported living in neighborhoods with higher residential density. Among participants aged 79–89 years, the subgroup (of three distinct subgroups identified) with the lowest obesity prevalence (19.4 %) consisted of non-African American/Black participants who reported living in neighborhoods with friends or acquaintances similar in demographic characteristics to themselves. Overall support for the partitioned subgroupings was obtained using mixed model regression analysis.

**Conclusions:**

The results suggest that, in combination with race/ethnicity, features of the perceived neighborhood built and social environments differentiated distinct groups of vulnerable older adults from different age strata that differed in obesity prevalence. Pending further verification, the results may help to inform subsequent targeting of such subgroups for further investigation.

**Trial registration:**

Clinicaltrials.gov Identifier = NCT01072500

## Background

Obesity represents one of the most pressing public health issues in an increasing number of countries around the world [1–4]. It is strongly linked with disability as well as life-threatening diseases such as cardiovascular disease (myocardial infarction, stroke, peripheral vascular disease), type 2 diabetes, and a number of cancers [5]. It previously has been argued that the association between obesity status and U.S. adult mortality risk lessens with age [6, 7]. However, a more recent examination of U.S. National Health Interview Survey data accounting for age-associated confounding (e.g., unequal cohort mortality; age-related survey selection bias) suggests that the obesity-mortality relationship may actually grow stronger with age [5].

U.S. adult men and women ages 60 years and above are today more than three times (for men) and 1.6 times (for women) as likely to be obese than their counterparts four decades ago [8, 9]. The overall aging of the U.S. population and the general rise in obesity prevalence across the population, including among older age groups, provides a strong rationale for a more comprehensive evaluation of factors of potential importance in contributing to obesity heading into late life [10].

Traditionally, obesity research in older age groups has focused on individual-level variables, such as age and psychosocial factors associated with eating behaviors [11]. Yet, among the putative factors prominently contributing to obesity rates across the life course are “obesogenic environments” that set the stage for higher average caloric intakes and fewer daily opportunities for walking, bicycling, and other forms of regular physical activity [12]. Such observations reflect an ecological model of obesity that recognizes the interplay of multi-level inputs, in particular, individual characteristics by environmental context interactions, underlying obesity [13].

The overall surge in research aimed at understanding the relationships between obesity levels and environmental contexts [14–16] has largely failed to include studies specifically targeting older adults [17, 18]. This is particularly the case for those at higher risk of functional decline and major mobility disability for whom obesity may be especially deleterious [19, 20]. Therefore, recognizing that obesity is a product of person by environmental factors [12, 21], the main objective of this investigation was to study the joint relations of sociodemographic and perceived built environment factors with the likelihood of being obese in an older adult population at risk for mobility disability. A substantial body of evidence is now available supporting the observation that perceived and objective environmental measures assess two distinct dimensions of the built environment [22–24], and that neighborhood perceptions may be more closely related to actual behaviors [25, 26]. In addition, systematically collected information on the relations between such perceived environment variables and obesity among older adults with some mobility impairments—who, in light of the aging of populations around the world are a growing segment of the older adult population---are particularly scarce.

The major question of interest at baseline was:

- Which perceived built environment variables, either alone or in combination with other more commonly measured sociodemographic variables, most efficiently and effectively delineated those subgroups of LIFE participants at increased likelihood of obesity?

## Methods

### Sample and procedures

The LIFE study sample and methods have been described previously [27]. Briefly, the major objective of this multi-center, single-blinded randomized trial was to evaluate the effects of a comprehensive physical activity program on the prevention of major mobility disability. The original sample consisted of 1635 adults who met the following major eligibility criteria [28]: ages 70–89 years; sedentary (reporting <20 minutes/week in the past month performing regular physical activity and less than 125 minutes/week of moderate-to-vigorous physical activity); at high risk for mobility disability based on lower extremity functional limitations measured by the Short Physical Performance Battery (SPPB) [29] score ≤9 out of 12 (45 % of participants were targeted to have a score <8); could walk 400 meters in ≤15 min without sitting, leaning, or the help of another person or walker; had no major cognitive impairment (Modified Mini-Mental State Examination [3MSE] [30] 1.5 standard deviations below education- and race-specific norms); and could safely participate in the intervention as determined by medical history, physical exam and resting ECG. The eight field centers were: University of Florida, Gainesville and Jacksonville, Florida; Northwestern University, Chicago, Illinois; Pennington Biomedical Research Center, Baton Rouge, Louisiana; University of Pittsburgh, Pittsburgh, Pennsylvania; Stanford University, Stanford, California; Tufts University, Boston, Massachusetts; Wake Forest School of Medicine, Winston-Salem, North Carolina; and Yale University, New Haven, Connecticut. The primary recruitment strategy was targeted community mass mailings. The institutional review boards at all participating sites approved the study protocol. Written informed consent was obtained from all study participants. The trial was monitored by a data and safety monitoring board appointed by the National Institute on Aging, and was conducted between February 2010 and December 2013.

### Measurements

#### Obesity

Obesity was measured objectively at baseline in each LIFE field center by centrally trained and certified study staff. Height was measured to the nearest cm using a wall-mounted stadiometer, and body weight was measured without shoes using a calibrated scale [31]. Body mass index (BMI) was calculated as weight (kg)/ height (m)^2^. Obesity was defined as BMI ≥ 30 [12].

#### Perceived built environment

Perceived built environment was assessed using the NEWS-A (24-item version). The NEWS-A is a validated measure of perceived built environment elements that has been used frequently in large-scale studies [32]. The perceived built environment domains captured by this measure have been found to be associated with physical activity levels and body weight in a range of populations, including geographically derived samples of young to middle age [14] and older adults [17]. Perceived built environment domains consisted of the main type of housing in each participant’s neighborhood, reflecting residential density (e.g., 5 alternatives, for example, detached single-family housing, or a mix of single-family residences and town houses, row houses, apartments or condos); access to services in the neighborhood (5 items); street connectivity (3 items); walkability (2 items) (e.g., presence of sidewalks); safety from crime (5 items); pedestrian/traffic safety (4 items) (e.g., presence of crosswalks and pedestrian signals); travel access (1 item) (i.e., in a typical week one can conveniently, safely and affordably travel to all the places that one would like, yes/no); aesthetics (2 items) (e.g., presence of trees and foliage along the streets); and seeing many others being physically active in the neighborhood (1 item). Except for the housing and travel access questions, all items were rated on a 4-point “strongly disagree” to “strongly agree” scale. For perceived built environment domains containing more than one item, a mean of the items was calculated.

Participants also completed a neighborhood social cohesion measure [33]. Examples of the 5 social cohesion items (rated on a 1–5 scale from “strongly disagree” to “strongly agree”) were: “people in the neighborhood are willing to help their neighbors”; and “people in the neighborhood do not share the same values”. Items on this measure were averaged for each participant. A sixth item included in this measure pertained to perceived intergenerational/socioeconomic neighborhood mix and affiliation (“I have acquaintances/friends in my neighborhood that differ from me in terms of age, income or ethnicity.”) This item taps into an aspect of homophily, i.e., the tendency of individuals to associate and develop ties with those perceived as similar to themselves [34, 35].

Each of the items in the baseline questionnaires had less than 1 % of responses missing except for the travel access question, which had a total of 4 % missing. For any variables containing more than one item, the summary variable was calculated as the mean of all non-missing items.

#### Sociodemographic, physical activity, and mobility-related variables

Self-reported sociodemographic variables were collected at baseline [28]. These included gender, educational attainment, income category, age, race/ethnicity (white, African American/Black, Hispanic, other), marital status (married, separated/divorced, widowed, never married), study site, and number living in household. We also collected a measure of self-reported health [27], self-reported walking and total physical activity levels using the Community Healthy Activities Model Program for Seniors (CHAMPS) questionnaire [36], and accelerometry measuring daily movement over a 7-day period [Actigraph Inc., Pensacola FL] [17]. Measured baseline mobility-related function was determined using the Short Physical Performance Battery (SPPB) [37] and the 400 meter walk test [38]. (See Fielding et al., 2011 for a detailed description of these measures) [27].

### Statistical analysis

#### Recursive partitioning analysis

To accomplish our goal, recursive partitioning analysis (i.e., signal detection analysis), which has been applied for decades in the medical field [39], was used to identify the combinations of baseline perceived environment and individual-level variables that best differentiated those participants who were obese at baseline (i.e., BMI of 30 and above) from those who were not [40]. The categorization of obesity was selected as the major variable of interest given its increasing prevalence in older adults [41] and its detrimental effects on health, day-to-day function, and quality of life as people age [41]. A recursive partitioning approach was chosen given its particular utility in identifying higher-order interactions that have previously not been well specified in the literature, and thus could be missed by regression approaches that require interaction terms to be specified up front. This analytic approach is thus well suited to this multi-level ecological exploration of vulnerable older adults. Other advantages of signal detection analysis include reduction of missing data problems among correlates because of independent evaluation of each variable in turn through application of receiver-operator curves (ROC); and the ability of this method to efficiently specify the actual cut-point for each variable above or below which the probability of having the health condition of interest (in this case, obesity) is most increased [42]. In some situations, this recursive partitioning approach has been shown to better identify distinct and homogeneous subgroups than regression methods [43]. The whole sample was included in the initial step in evaluating each cut-point of each variable to determine which variable cut-point resulted in the maximal differentiation of percentage obese, resulting in two subgroups. This process of determining which variable cut-point produced the maximum differentiation of percentage obese was repeated for each new subgroup found until the stopping rules, below, ended the analysis.

The stopping rules included no evaluation with fewer than 100 participants at any variable step of the recursive partitioning process [44], and no fewer than 90 participants in a subgroup. A significance level of *p* < .05 was applied at each step. Perceived environment subscale values were rounded to the nearest decimal (.1, .2, etc.). Individual-level variables such as BMI were evaluated in integer-based increments, starting with the lowest value of the dataset.

The recursive partitioning analysis was conducted using a two-step approach. The first step consisted of evaluating significant differences in the occurrence of the health condition of interest (obesity) by key sociodemographic variables (e.g., age, gender, race/ethnicity), to determine if a stratified analysis was indicated. This initial signal detection analysis consisted of the sociodemographic variables of age, gender, race/ethnicity, education, income, marital status, study site location, number of people in the household, and living alone. Based on this analysis, age was identified as the most efficient partitioning variable differentiating the sample into subgroups at higher or lower obesity levels. The most efficient age cut-point that was most strongly associated with the probability (inverse, in this case) of obesity was age 79 years or older versus less than 79 years (*X*^*2*^ = 123, *p* < .0001). Therefore, for the second step of the analysis, we stratified the sample at this age cut-point and explored the inclusion of perceived environment correlates of obesity in each of these two age-derived strata.

The perceived environment variables included in the second age-stratified recursive partitioning step were perceived residential density, access to services, street connectivity, walkability, travel access, aesthetics, crime, pedestrian/traffic safety, observing others engaging in physical activity in the neighborhood, neighborhood social cohesion, and neighborhood intergenerational/SES mix and affiliation. We also included the sociodemographic variables that were part of the step 1 recursive partitioning analysis described above. As part of the study eligibility criteria, participants had to be insufficiently active with presence of some lower-extremity/mobility limitations to be enrolled [27]. An objective measure of physical activity—accelerometry—was used to assess average movement throughout the day including in moderate to vigorous activity, and average walk time via a 400-meter walk test reflected mobility limitations. Because these two variables were strongly correlated at baseline (e.g., Spearman Rho = −.42, *p* < .0001), and daily physical activity level is an important behavioral public health target for obesity control as well as for numerous other health conditions associated with aging [12, 45], we included the accelerometry-derived physical activity variable in the signal detection models. (As described below, potential subgroup differences in mobility limitations were explored as part of the descriptive profile analyses that were conducted following the recursive partitioning analyses).

#### Subgroup profiles

As is typically performed with this type of recursive partitioning method [42, 43, 46], further descriptive analysis was conducted on the distinct subgroups identified through the age-stratified recursive partitioning analyses in order to better understand subgroup membership. All variables entered into the recursive partitioning analyses were evaluated, in addition to the initial screening variables for self-reported physical activity (CHAMPS questionnaire measuring total physical activity, total walking, and walking for errands variables [36]—the latter two variables being most typically associated with built environment features [47–50]), and lower-extremity function measured via the Short Physical Performance Battery (SPPB) [37]. Analysis of variance was used to determine whether any differences between subgroups were apparent, followed by pair-wise comparison testing for those variables reaching statistical significance to identify where the between-group differences lay. Alpha was set at p ≤ .01 in exploring between-group differences in these subgroup profiles to aid interpretation of the results.

#### Multilevel regression analysis to provide further information on the magnitude of the associations identified through recursive partitioning

To further delineate the magnitude of the associations accompanying the recursive partitioning method, a series of multilevel regression models were completed using each partitioning variable as the independent variable, and adjusting for effects of the other covariates. For each partitioning variable used, we modeled BMI as a continuous variable (mixed effects linear regression, with study site included as a random effect) (SAS’s PROC MIXED) [51] and as a dichotomous variable (obese vs. non-obese) (generalized linear mixed model invoking a logit link function) (SAS’s PROC GLIMMIX) [52]. For the models using BMI (continuous) as the dependent variable, the regression estimates for the independent variables (partitioning variables) represent how many BMI units were essentially gained or lost in association with a given variable (e.g., race, education, built environment features, etc.). For the dichotomous dependent variable regression models, we generated an estimate of the odds of being obese for each partitioning variable. For the first identified partitioning variable, the model was based on the full sample at the given age stratum. For subsequent partitioning variables (independent variables for each model), the sample was stratified based on the splits identified by the recursive partitioning analysis. This approach was preferred over the use of interaction terms (using a single model based on the full sample), since it allows for the inclusion of different covariates per model. This was important to avoid collinearity issues that arose when including all of the partitioning variables identified by the signal detection analysis in the same model. Collinearity was assessed using Variance Inflation Factors (VIF), such that multicollinearity was determined if VIF > 10, in which case the only variable kept in the final model was the one explaining the greatest variability in the dependent variable. This was the case for education and income, which were strongly correlated, and, therefore, only education was kept in the final models. The same scenario occurred for residential density, street connectivity, access to destinations, and safety from crime (only residential density was kept in the final models). All analyses were performed using SAS 9.3 (SAS Institute, Cary, NC). The current investigation is a “hypothesis-generating” exploratory study to inform future investigations; thus, caution should be used in interpreting the large number of tests that were conducted.

## Results

### Sample description

Descriptive statistics for the LIFE sample are summarized in Table 1. Sixty-seven percent were women, and 24 % were of nonwhite race or Hispanic ethnicity. The average age of the sample was 78.9 ± 5.2 years. Mean measured baseline BMI was 30.2 ± 6.1 (mean and standard deviation for men = 30.1 ± 5.8; for women = 30.3 ± 6.2). The median baseline BMI for the sample was 29.4. Descriptive information related to the baseline perceived environmental variables is summarized in Table 2 by site. Thirty-five of the original 1635 LIFE participants failed to complete the environmental questionnaire. The current investigation focuses on the remaining 1600 participants.

**Table 1:** LIFE trial baseline descriptive statistics

	All		Age ≤ 78		Age ≥ 79	
Variable	N	%	N	%	N	%
Gender						
Female	1098	67.2	585	69.5	513	64.7
Male	537	32.8	257	30.5	280	35.3
Race						
White	1239	75.8	582	69.1	657	82.8
African American/Black	288	17.6	189	22.4	99	12.5
Hispanic	61	3.7	42	5.0	19	2.4
Other	47	2.9	29	3.4	18	2.3
Marital Status						
Married	583	35.9	329	39.1	254	32.3
Separate/divorced	268	16.5	185	22.0	83	10.6
Widowed	661	40.6	257	30.5	404	51.5
Never married	101	6.2	60	7.1	41	5.2
Other	13	0.8	10	1.2	3	0.4
Education						
No formal education	13	0.8	8	0.9	5	0.6
Elementary	31	1.9	13	1.6	18	2.3
High school	484	29.7	253	30.1	231	29.3
College	642	39.4	322	38.3	320	40.6
Postgraduate	402	24.7	214	25.4	188	23.8
Other	58	3.5	31	3.7	27	3.4
Household Income						
Less than $10,000	67	4.6	33	4.5	34	4.9
$10,000 to $14,999	113	7.8	68	9.0	45	6.6
$15,000 to $24,999	282	19.5	144	19.0	138	20.1
$25,000 to $34,999	222	15.4	113	14.9	109	15.9
$35,000 to $49,999	288	19.9	148	19.6	140	20.4
$50,000 to $74,999	233	16.1	120	15.9	113	16.4
$75,000 or greater	239	16.6	131	17.1	108	15.7
Health self rating						
Excellent	105	6.4	44	5.2	61	7.7
Very good	462	28.4	240	28.6	222	28.1
Good	791	48.6	403	48.0	388	49.1
Fair	251	15.4	141	16.8	110	14.0
Poor	20	1.2	12	1.4	8	1.0
Obesity-Class I [BMI 30–34.9]	434	26.5	254	30.2	180	22.7
Obesity-Class II [BMI 35–39.9]	202	12.4	145	17.2	57	7.2
Obesity-Class III [BMI ≥ 40]	116	7.1	100	11.9	16	2.0
Variable	Mean	SD	Mean	SD	Mean	SD
Age (yr.)	78.9	5.2	74.5	2.5	83.5	2.9
Body Mass Index	30.2	6.0	32.0	6.4	28.3	4.8
Number in household	1.7	1.0	1.8	1.0	1.6	0.9

**Table 2:** Means and standard deviations of perceived built environment variables, by site*

	Site								
Variable (range of values^**^)	Northwestern	LSU	Stanford	Tufts	Florida	Pittsburgh	Wake Forest	Yale	Overall
(N)	195	208	198	192	191	213	203	200	1600
Residential density (1–5)	2.73(1.61)^bcdefgh^	1.21(0.56)^adfh^	1.40(0.80)^adf^	2.14(1.21)^abcefgh^	1.38(0.84)^adfh^	1.70(1.06)^abcdeg^	1.41(0.76)^adf^	1.65(0.99)^abde^	1.70(1.12)
Access to services (1–4)	3.00(0.69)^bcdefgh^	2.21(0.54)^acdg^	2.67(0.64)^abefgh^	2.69(0.75)^abefgh^	2.33(0.62)^acdgh^	2.32(0.70)^acdgh^	2.04(0.68)^abcdef^	2.07(0.71)^acdef^	2.41(0.74)
Travel access (0–1)	0.83(0.38)^b^	0.95(0.23)^afh^	0.89(0.32)	0.86(0.35)	0.90(0.30)	0.82(0.39)^b^	0.87(0.34)	0.83(0.38)^b^	0.87(0.34)
Street connectivity (1–4)	3.25(0.67)^bcefgh^	2.78(0.82)^ad^	2.91(0.71)^aeg^	3.11(0.69)^befgh^	2.71(0.89)^acdf^	2.91(0.77)^adeg^	2.70(0.84)^acdf^	2.75(0.77)^ad^	2.89(0.79)
Walkability (1–4)	3.48(0.76)^bcdefgh^	2.28(1.06)^acdeg^	2.97(1.09)^abefgh^	3.14(0.92)^abefgh^	2.01(1.03)^abcdf^	2.54(1.23)^acdegh^	1.79(1.04)^abcdfh^	2.14(1.17)^acdfg^	2.54(1.19)
Aesthetics (1–4)	3.32(0.66)^bfgh^	3.00(0.88)^acde^	3.37(0.64)^bfgh^	3.33(0.62)^bfgh^	3.31(0.69)^bfgh^	2.92(0.79)^acde^	2.98(0.85)^acde^	3.10(0.74)^acde^	3.16(0.76)
Traffic safety (1–4)	2.84(0.59)^befgh^	2.45(0.70)^acde^	2.84(0.59)^befgh^	2.78(0.58)^bfgh^	2.67(0.64)^abcgh^	2.53(0.63)^acdgh^	2.33(0.70)^acdef^	2.34(0.63)^acdef^	2.59(0.66)
Crime safety (1–4)	3.00(0.64)^bcd^	3.25(0.66)^af^	3.29(0.52)^afh^	3.30(0.53)^afh^	3.14(0.68)^f^	2.98(0.60)^bcdeg^	3.16(0.61)^f^	3.13(0.57)^cd^	3.16(0.61)
Social cohesion (1–5)	3.45(0.75)^bcdegh^	4.03(0.76)^acdefgh^	3.77(0.73)^ab^	3.72(0.72)^ab^	3.83(0.82)^abf^	3.62(0.73)^beg^	3.83(0.81)^abf^	3.66(0.73)^ab^	3.74(0.77)
Social modeling (1–4)	3.14(0.92)^bfgh^	2.86(1.04)^adfg^	3.04(0.95)^fgh^	3.14(0.89)^bfgh^	2.93(1.01)^fgh^	2.55(1.07)^abcde^	2.53(1.09)^abcde^	2.66(1.01)^acde^	2.85(1.03)
Intergenerational mix (1–5)	3.83(1.12)	3.87(1.16)	3.87(1.16)	3.91(1.13)	3.86(1.14)	3.71(1.14)	3.61(1.25)	3.67(1.20)	3.79(1.16)

*P values for omnibus tests of significance (ANOVA) set at ≤ .01. Superscripts indicate significantly different groups for those variables in which the omnibus test reached statistical significance

^**^Higher score denotes a favorable outcome for each variable

### Recursive partitioning analysis and multilevel regression estimates

#### Less than 79 years age stratum

**Fig. 1:**
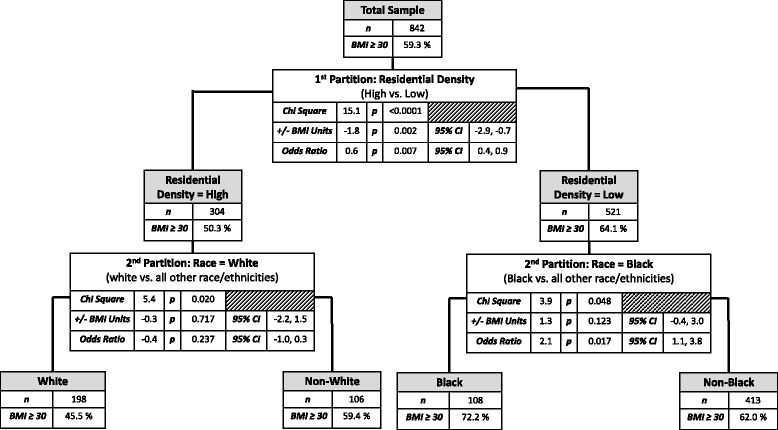
Results of the recursive partitioning analysis for participants aged < 79 years

The results of the recursive partitioning analysis for this age stratum and the corresponding mixed effects regression estimates (providing the magnitude of the association between each identified independent variable and BMI, as well as the odds of being obese in association with each independent variable) are summarized in Fig. 1. Fifty-nine percent of the overall sample had a BMI ≥ 30 (i.e., were obese). The recursive partitioning analysis identified four distinct subgroups that differed significantly on the likelihood of obesity. The variable that best identified the subgroup most likely to be obese was residential density. In the subgroup which reported lower levels of residential density (i.e., neighborhoods containing primarily detached, single-family housing) (n = 521), 64.1 % were obese, relative to 50.3 % obese in the subgroup reporting greater residential density (i.e., a mix of single-family housing, apartments, and townhouses), *X*^*2*^ = 15.07, *p* < .0001. The results from the mixed effects regression models showed that reporting more residential density was associated with having 1.8 less BMI units relative to reporting less residential density (95 % CI: −2.9, −0.7; p = .002), and the odds of being obese were 40 % lower (OR = 0.6, 95 % CI: 0.4, 0.9; p = 0.007) for those reporting more residential density than for participants reporting less density.

The recursive partitioning analysis partitioned both residential density subgroups further based on reported race/ethnicity, resulting in a total of four distinct subgroups that differed significantly in their obesity prevalence (Fig. 1). The subgroup with the lowest obesity levels in this age stratum (45.5 %) reported greater residential density in combination with being of white non-Hispanic race/ethnicity. In contrast, the subgroup reporting similar levels of residential density but non-white race/ethnicity had a significantly higher proportion of obese participants (59.4 %), *X*^*2*^ = 5.40, *p* = .02). For this latter subgroup only, the results of the adjusted mixed effect regression models estimating the magnitude of the association showed no significant association between residential density and BMI/obesity among this non-white race/ethnicity subgroup (Fig. 1). We also ran unadjusted models (data not shown) that showed significant associations for residential density and BMI/obesity for both racial/ethnic subgroups, consistent with the recursive partitioning analysis (*p* < 0.05), but statistical power was lost when introducing the adjusting variables.

For participants reporting lower residential density levels, those reporting being non-African American/Black had a significantly lower proportion of obese participants (62.0 %) relative to those reporting being African American/Black (72.2 %), *X*^*2*^ = 3.90, *p* = .048. These results were supported by the mixed effects logistic regression model: African American/Black older adults who reported living in neighborhoods with low residential density had 2.1 higher odds of being obese than non-African American/Black participants reporting living in neighborhoods with low residential density (95 % CI: 1.1, 3.8; *p* < .017).

#### 79 or greater years age stratum

**Fig. 2:**
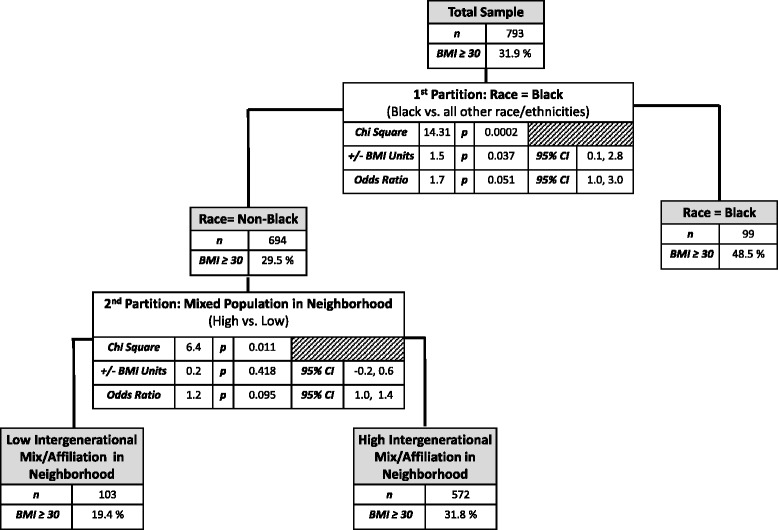
Results of the recursive partitioning analysis for participants aged ≥ 79 years

Figure 2 provides the results of the recursive partitioning analysis for the older age strata and the corresponding mixed effects regression estimates. Thirty-two percent of the overall sample had a BMI ≥ 30 (i.e., were obese). The recursive partitioning analysis identified three distinct subgroups that differed significantly on the likelihood of obesity. The variable that best identified the subgroup most likely to be obese was African American/Black race. In the African American/Black subgroup (*n* = 99), 48.5 % were obese, relative to 29.5 % obese in the non-African American/Black subgroup, *X*^*2*^ = 14.31, *p* = .0002. The results from the mixed effects regression models showed that African American/Black race was associated with having 1.5 more BMI units relative to other races/ethnicities (95 % CI: 0.1, 2.8; *p* = .037), and, the odds of being obese were 1.7 times higher (95 % CI: 1.0, 3.0; *p* = 0.05) for those reporting being of African American/Black heritage relative to others.

The recursive partitioning analysis partitioned the non-African American/Black subgroup further, based on whether or not participants reported living in neighborhoods with friends or acquaintances in the neighborhood who did not differ in demographic characteristics (age, income, or ethnicity) from themselves, resulting in a total of three distinct subgroups that differed significantly in their obesity likelihood (Fig. 2). The subgroup with the lowest obesity levels (19.4 %) reported having friends or acquaintances in the neighborhood that did not differ in demographic characteristics from themselves (*n* = 103). In contrast, the non-African American/Black subgroup reporting being neutral to (20 %) or agreeing with (80 %) the statement concerning having friends or acquaintances in the neighborhood that differed in age, income, or ethnicity had a significantly higher proportion of obese participants (31.8 %; *n* = 572), *X*^*2*^ = 6.40, *p* = .01).

The results from the fully adjusted mixed effect logistic regression model was marginally significant, likely due to the reduced sample size within this sub-stratum, but was consistent in terms of direction with the results of the recursive partitioning analysis: i.e., among non-African American/Black participants in this older age strata who reported having friends or acquaintances in the neighborhood who differed in demographic characteristics from themselves, the odds of being obese were 1.2 higher than for those not reporting this type of neighborhood situation (95 % CI: 1.0, 1.4; *p* = .095).

### Subgroup profiles

Further descriptive analysis was conducted on the four distinct subgroups in the lower age stratum and the three distinct subgroups in the higher age stratum identified through the recursive partitioning analysis to better understand subgroup membership. The following descriptive differences were noted (see Tables 3 and 4; *p* values ≤ .01 for these analyses):

**Table 3:** Summary profiles for signal detection subgroups, age less than 79 years*

Baseline variables	Signal detection subgroup			
	(A) *n* = 198	(B) *n* = 106	(C) *n* = 413	(D) *n* = 108
	Resid. density high, White non-Hispanic	Resid. density high, non- White	Resid. density lower, non- Black	Resid. density lower, Black
	(45.5 % obese)	(59.4 % obese)	(62.0 % obese)	(72.2 % obese)
% Male	33.8^bd^	17.0^ac^	36.3^bd^	14.8^ac^
Age (yrs; mean)	74.1^d^	73.9	74.3^d^	73.3^ac^
Education (mean)	4.2^bcd^	3.8^a^	3.9^a^	3.9^a^
Income (mean)	5.5^bc^	4.6^acd^	5.9^abd^	5.2^bc^
% Live alone	61.6^cd^	59.4^cd^	38.7^ab^	33.3^ab^
% African American/Black	0.0^bd^	74.5^acd^	0.0^bd^	100.0^abc^
% White	100.0^bcd^	0.0^ac^	90.1^abd^	0.0^ac^
% Hispanic	0.0^bc^	15.1^acd^	5.8^abd^	0.0^bc^
% Other race/ethnicity	0.0^bc^	11.3^acd^	4.1^abd^	0.0^bc^
% Married	29.8^bc^	16.0^acd^	51.3^abd^	33.3^bc^
% Separated/divorced	24.8^bc^	38.7^acd^	14.5^abd^	25.0^bc^
% Widowed	31.3	34.0	28.1	37.0
% Never married	12.63^cd^	10.38^cd^	4.60^ab^	3.70^ab^
Number in household (mean)	1.5^bcd^	1.8^ad^	1.9^a^	2.1^ab^
Self rated health (mean)	2.7^bd^	3.1^ac^	2.7^bd^	3.1^ac^
Residential density (mean)	2.9^bcd^	2.7^acd^	1.0^ab^	1.0^ab^
Access to services (mean)	2.8^cd^	2.8^cd^	2.2^abd^	2.4^abc^
Travel access (mean)	0.9	0.8	0.9	0.9
Street connectivity (mean)	3.0^c^	3.1^c^	2.7^abd^	3.1^c^
Walkability (mean)	3.1^cd^	3.1^cd^	2.1^abd^	2.6^abc^
Aesthetics (mean)	3.3	3.0	3.2	2.8
Pedestrian/Traffic safety (mean)	2.7^bcd^	2.6^a^	2.5^a^	2.6^a^
Safety from crime (mean)	3.1^bd^	2.9^ac^	3.2^bd^	2.9^ac^
Social cohesion (mean)	3.6^c^	3.4^cd^	3.8^ab^	3.7^b^
Social modeling^†^ (mean)	3.1^bcd^	2.6^a^	2.8^ad^	2.5^ac^
Intergenerational/socioeconomic neighborhood mix & affiliation (mean)	3.8	3.7	3.8	3.7
Body Mass Index [BMI] (mean)	30.3^cd^	31.6	32.5^a^	33.3^a^
SPPB total score^§^ (mean)	7.5	7.5	7.7	7.7
400 meter walk total time (seconds) (mean)	478.9	507.9	489.1	502.4
Accelerometer minutes with activity >100^#^ (mean)	190.9	211.9	202.5	206.5
CHAMPS baseline total activity min/week (mean)	443.0	509.0	470.7	428.5
CHAMPS baseline total walking min/week (mean)	162.3^cd^	148.2^cd^	111.8^ab^	81.4^ab^
CHAMPS baseline walking errands min/week (mean)	85.3^cd^	71.2^cd^	44.0^ab^	27.5^ab^

*P values for omnibus tests of significance (ANOVA for continuous outcomes, Chi-square for dichotomous outcomes) set at ≤ .01. Superscripts indicate significantly different groups for those variables in which the omnibus test reached statistical significance

^†^Social modeling = observing others walking or cycling in one’s neighborhood

^§^SPPB = Short Physical Performance Battery

^#^ *n* = 1306, due to delayed start in accelerometry data collection and elimination of data with technical errors

**Table 4:** Summary profiles for signal detection subgroups, age 79 years or greater*

Baseline variables	Signal detection subgroup		
	(A) *n* = 103	(B) *n* = 572	(C) *n* = 99
	Not Black, neighborhood friends do not differ in demo.	Not Black, neighborhood friends differ in demo.	Black
	(19.4 % obese)	(31.8 % obese)	(48.5 % obese)
% Male	35.0^c^	38.5^c^	18.2^ab^
Age (mean)	83.4	83.1	82.3
Education (mean)	3.9	4.0	3.9
Income (mean)	5.5^c^	5.6^c^	4.9^ab^
% Live alone	49.5	53.7	54.6
% African American/Black	0.0^c^	0.0^c^	100.0^ab^
% White	92.2^c^	95.1^c^	0.0^ab^
% Hispanic	4.9	2.5	0.0
% Other race/ethnicity	3.9	2.5	0.0
% Married	41.8	32.0	25.3
% Separated/divorced	9.7	9.1	17.2
% Widowed	42.7	52.1	53.5
% Never married	5.8	5.1	4.0
Number in household (mean)	1.6	1.6	1.7
Self rated health (mean)	2.7^c^	2.7^c^	3.0^ab^
Residential density (mean)	1.8	1.7	1.8
Access to services (mean)	2.3	2.4	2.5
Travel access (mean)	0.8	0.9	0.8
Street connectivity (mean)	2.8	2.9	3.1
Walkability (mean)	2.3^c^	2.5^c^	3.0^ab^
Aesthetics (mean)	3.2^c^	3.3^c^	2.9^ab^
Traffic safety (mean)	2.7	2.6	2.6
Safety from crime (mean)	3.3^c^	3.2^c^	3.0^ab^
Social cohesion (mean)	3.9	3.8	3.6
Social modeling^†^ (mean)	2.7^b^	3.0^ac^	2.6^b^
Intergenerational/socioeconomic neighborhood mix & affiliation (mean)	1.5^bc^	4.2^a^	3.8^a^
Body Mass Index [BMI] (mean)	27.2^bc^	28.4^ac^	29.9^ab^
SPPB total score^§^ (mean)	7.0	7.2	6.9
400 meter walk total time (seconds) (mean)	529.0^c^	518.7^c^	563.4^ab^
Accelerometer minutes with activity >100^#^ (mean)	172.4	171.3	177.5
CHAMPS baseline total activity min/week (mean)	465.9	526.3	426.4
CHAMPS baseline total walking min/week (mean)	104.1^b^	142.2^ac^	93.0^b^
CHAMPS baseline walking errands min/week (mean)	31.0	53.6	39.1

*P values for omnibus tests of significance (ANOVA for continuous outcomes, Chi-square for dichotomous outcomes) set at ≤ .01. Superscripts indicate significantly different groups for those variables in which the omnibus test reached statistical significance

^†^Social modeling = observing others walking or cycling in one’s neighborhood

^§^SPPB = Short Physical Performance Battery

^#^ *n* = 1306, due to delayed start in accelerometry data collection and elimination of data with technical errors

#### For the ≤79 age stratum

- **Subgroup A** (i.e., white non-Hispanic participants reporting living in neighborhoods with the highest residential density) had the lowest percentage of obese participants in this age stratum (45.5 %), the lowest mean body mass index (mean = 30.3 ± 6.6) the highest mean education level, a greater percentage of men (33.8 %), the lowest mean number of people living in their household, and relatively high levels of self-rated health. Subgroup members reported living in neighborhoods with elements most conducive to active living, including access to services, neighborhood walkability, observing others walking or cycling in the neighborhood (i.e., social modeling), safety from crime, and pedestrian/traffic safety. This subgroup also reported the highest mean total walking minutes/week and mean minutes walking for errands/week (see Table 3).
- **Subgroup B** (i.e., non-white participants reporting living in neighborhoods with higher residential density), which had the second lowest percentage of obese participants in this age stratum (59.4 %), also reported having some neighborhood elements conducive to active living, including access to services, and neighborhood walkability. However, this subgroup reported significantly lower levels than Subgroup A of other “active living” elements, including pedestrian/traffic safety, safety from crime, and observing others walking or cycling in the neighborhood (social modeling). They also reported lower mean levels of self-rated health, a greater number of people in the household, a higher proportion of women, lower levels of education and income, and a lower percentage that were married relative to the other three subgroups in this age stratum. Similar to Subgroup A, this subgroup reported higher mean total walking minutes/week and mean minutes walking for errands/week relative to the two subgroups living in lower residential density neighborhoods.
- **Subgroup C** (lower reported residential density, not African American/Black; 62 % obese) had reasonably similar sociodemographic characteristics and similar perceptions related to feeling safe from crime as Subgroup A, while reporting a smaller percentage of participants living alone than Subgroups A and B and the largest percentage of married participants overall. However, this subgroup had poorer ratings for neighborhood elements associated with active living, including access to services, street connectivity, neighborhood walkability, and observing others walking or cycling in the neighborhood, and lower mean total walking minutes/week and mean minutes walking for errands/week relative to Subgroups A and B.
- **Subgroup D** (lower reported residential density, African American/Black; 72 % obese) had the highest percentage of obese participants in this age stratum and the highest mean BMI. While reasonably similar to Subgroup B in sociodemographic characteristics such as educational level, race/ethnicity, a higher proportion of women, self-rated health, and feeling less safe from crime, they reported the lowest access to services in their neighborhood as well as low levels of neighborhood walkability. This subgroup also reported the lowest mean total walking minutes/week and mean minutes walking for errands/week.
  Among the descriptive variables that did not differ significantly between the four subgroups in this age stratum were SPPB score, total 400 meter walk time, accelerometer minutes, and CHAMPS total activity minutes/week (all *p* values ≥ .08).

#### For the 79± age stratum

**Subgroup A** (i.e., participants who were not African American/Black and reported friends or acquaintances in their neighborhood who do not differ from them in terms of age, income or ethnicity) shared a number of socioeconomic and perceived environmental characteristics with **Subgroup B** (participants who were not African American/Black and reported friends or acquaintances in their neighborhood that differed from them in terms of age, income or ethnicity) that distinguished them from **Subgroup C** (African American/Black race). These characteristics included a higher proportion of men, higher reported income level, better self-rated health, and higher perceived safety levels from crime. They also had a lower (better) 400-meter walk test time than Subgroup C. In terms of perceived environment characteristics, Subgroups A and B reported a higher level of neighborhood aesthetics, though less “walkability” (defined in the urban planning/community design literature as having structural characteristics more similar to urban than suburban areas) [18] than Subgroup C.

Subgroup A participants reported more homophilous friends or acquaintances in their neighborhoods (individuals more similar to themselves) than participants in either Subgroups B or C. Surprisingly, however, while Subgroup A had the smallest proportion of obese participants as well as the lowest BMI mean levels relative to the other subgroups in this and the younger-aged stratum, the mean total minutes of walking/week reported by this subgroup was significantly lower than that reported by Subgroup B (who lived in neighborhoods with higher perceived levels of walkability). Subgroup B also reported observing a greater number of others walking or cycling in their neighborhoods relative to Subgroups A or C.

Among the descriptive variables that did not differ significantly between the subgroups in the older-aged stratum were SPPB total score, accelerometer minutes, and mean CHAMPS total activity and walking for errands minutes per week (all *p* values ≥ .09).

## Discussion and conclusions

This investigation adds to current information on perceived environmental contexts and obesity by focusing on a vulnerable population segment—older adults at heightened risk for mobility disability—for which obesity plays a major role for health and function, but which has received relatively less attention in this area than working-age populations. The results, commensurate with an ecological model for understanding obesity [12], suggest that perceived built and social environmental features in combination with demographic variables can successfully differentiate subgroups of older adults with higher and lower obesity prevalence. The size of the LIFE older adult sample (1600 adults between the ages of 70 and 89 years) allowed us an opportunity to explore combinations of variables associated with obesity in two older age strata identified by the recursive partitioning method as most distinct with respect to obesity prevalence. In both age strata (70–78 years and 79–89 years of age), race/ethnicity was an important differentiator of obesity prevalence; white non-Hispanic participants had a lower prevalence of obesity relative to non-white participants in the younger age stratum (70–78 years), and non-African American/Black participants in the older age stratum had a lower prevalence of obesity than African American/Black participants in that age stratum. As indicated in the profile analyses, participants identifying themselves as part of a racial/ethnic minority group generally reported having fewer neighborhood elements typically associated in other age groups with lower obesity levels, such as less safety from crime and traffic. The results indicate that, similar to younger age groups [53], racial/ethnic minority characteristics are associated with aspects of the perceived neighborhood environment that may impede obesity-relevant health-promoting behaviors in physically vulnerable older adults as well.

In analyses targeting each age stratum, race/ethnicity characteristics interacted with aspects of the perceived neighborhood environment in identifying distinct subgroups with higher or lower probabilities of obesity. In the 70–78 year age stratum where obesity is most prevalent (59.3 %, vs. 31.9 % among participants 79 years or older), residential density, an important aspect of the built environment, was found to be the most potent variable distinguishing higher from lower obese subgroups. The residential density results are consistent with those from a geographically derived sample of older adults living in two U.S. regions and representing a range of mobility-related abilities [17].

Living in higher density neighborhoods (i.e., with a mix of single and multi-family housing) was associated in the 70–78 year age stratum with lower obesity rates for white non-Hispanic participants. In contrast, living in lower density neighborhoods (primarily detached single family housing) was associated with higher obesity rates in particular for African American/Black participants. The obesity rates in the latter subgroup were almost 30 % higher than in the former subgroup. Among the other baseline characteristics from the descriptive profile analyses that were significantly lower in the African American/Black subgroup living in lower density neighborhoods were low access to services in the neighborhood, lower neighborhood walkability, and the lowest average total walking minutes and minutes walking for errands per week. Such variables have been associated with obesity in a Census-derived sample of older adults drawn from two U.S. regions [17]. Given, as found in this investigation as well as others, older African Americans/Blacks are at particular risk for obesity, developing multi-level intervention strategies that span levels of potential impact (e.g., individual, environmental) is strongly indicated. This may be particularly the case for those older African Americans/Blacks living in less residentially dense neighborhoods.

In contrast, in the 79–89 year age stratum, a social environment characteristic, i.e., reporting having friends or acquaintances in the neighborhood that did not differ from oneself in age, income, or ethnicity, was associated, in combination with non-African American/Black race/ethnicity, with the lowest levels of obesity found in the current investigation (19 %--a 2 to 3-fold lower rate than other subgroups identified from the analyses). This variable reflects a principle of homophily, which suggests that people’s social ties of virtually every type (including neighborhood ties) tend to be homogeneous with respect to sociodemographic, behavioral, and intrapersonal characteristics [35, 54]. The current results suggest that such homophilous relationships among the very old may in some way set the stage for conditions or behaviors linked with more healthful weight, as has been found in prior online social network studies of younger adults [34]. While in a prospective study the spread of obesity through social ties was not found for neighbors living in the same geographic locale [55], the population under study was generally younger than in the current investigation. In a study of social networks and obesity in Montreal, researchers showed among older adults that having close neighborhood friends who exercised helped reduce their own risk of obesity by reducing their level of physical inactivity [56]. While results from our descriptive profile analyses did not support a physical activity mechanism underlying the lower proportion of obesity in the subgroup reporting more homophilous neighborhood relationships, it is possible that potential links between homophilous relationships and weight also could occur through dietary pathways, commensurate with an online social network study targeting a dietary behavior in younger adults [34]. Unfortunately, dietary information was not collected in the LIFE trial. Such conjectures await further research.

Interestingly, the subgroup in the 79+ age stratum reporting the highest levels of total physical activity and walking for errands per week was the one that reported having non-homophilous (more diverse) friends or acquaintances in their neighborhood. Previous research in a general adult population supports this finding, having shown that adults with greater network diversity were less likely to be inactive [57]. This subgroup also reported a less “walkable” neighborhood, relative to the subgroup reporting the least amount of total walking per week. Other investigations of perceived walkability and physical activity in older adults have reported more physical activity among some older adults living in less urban, more residential neighborhoods [58]. One possibility for this finding is that the greater presence of traffic and noise and lessened aesthetics (e.g., less pleasant scenery) in urban neighborhoods may serve as deterrents in the oldest age groups, particularly in those with some mobility impairment. Notably, the most active subgroup also reported the highest levels of seeing others walking or cycling in their neighborhood—a form of positive social modeling that has been reported in other studies of midlife and older adults [58, 59].

Walking is the physical activity behavior that has been linked most often to built environment features, and differences in reported walking were observed among the subgroups identified in the recursive partitioning analyses [48–50]. In contrast, levels of total daily movement and regular physical activity, measured using accelerometry and the CHAMPS questionnaire, respectively, did not significantly discriminate between the subgroups. Such activity measures assess additional aspects of physical activity that may have little to do directly with neighborhood features (e.g., activity occurring inside the home or at a community venue) [48]. It is also possible that, given the sample’s initially reduced levels of general physical activity set by the study exclusion criteria, we had insufficient overall variability in these baseline variables to detect differences.

The physically vulnerable older adult sample under study had a higher proportion of obesity (46 %) than nationally representative samples of older adults in this age group [10]. This is likely due to the trial’s physical inactivity and mobility impairment entry criteria.

By reporting BMI units gained or lost in association with residential density, race, and having friends or acquaintances in one’s neighborhood with different sociodemographic characteristics, we could confirm two observations: 1) that the recursive partitioning analysis successfully identified statistically distinct subgroups with respect to obesity rates for all cases but one; and 2) that the associations of the partitioning variables with BMI and/or obesity were both statistically strong and clinically meaningful (e.g., greater than 1 BMI unit). Such BMI differences have been estimated to result in a 17 % increase in heart failure incidence among the general population, and have been associated with elevated blood pressure among older adults [60, 61].

Among the strengths of the current work are its focus on a sample of community-dwelling older adults at risk for disability who have been little studied in the built environment field; study enrollment across eight regionally distinct field centers that enhanced diversity with respect to perceived built environment features; clinical assessment of body weight and physical function; and a sufficient sized older adult sample to allow for stratification by age—an important factor associated with body weight among older populations that has been insufficiently studied given the relative dearth of older adults in many built environment studies in the health promotion field. In addition, the large sample allowed for application of an innovative nonparametric statistical method for investigating the combination of factors linked with an important and clinically meaningful dichotomous health condition (presence or absence of obesity). This method, unlike regression models, allows for the identification of specific subgroups, described by concrete variable cut-points, that can more readily inform subsequent decision-making around population targets [62]. It also provides a solution to the collinearity problem that typically prevents correlated variables of interest from being studied simultaneously in a traditional regression model. The recursive partitioning approach was strengthened through subsequently using mixed effect models to determine effect sizes within the stratified data after the recursive portioning technique identified the most meaningful splitting variables.

With respect to limitations, because of its cross-sectional nature, temporal relations between obesity levels and perceived built environmental features cannot be ascertained. The baseline data in this large study of older adults were investigated specifically to ascertain the extent to which there were “naturally occurring” associations between a diverse set of variables and the presence of obesity in this population prior to the introduction of an intervention. In addition, the LIFE trial did not collect information on dietary patterns, preventing the exploration of the relations between dietary behavior, perceived built environment elements, and obesity. A proposed next step in this line of research would be to explore potential linkages between built and social environmental features and changes in weight and related health behaviors (i.e., physical activity levels, dietary patterns) over time. Of interest, recently published research on a multi-site sample of midlife and older U.S. adults suggests that increases in specific built environment features, such as residential density and density of walking destinations over time, were related to 10-year decreases in objective measures of BMI and waist circumference [16]. The number of baseline variables of interest in the recursive partitioning analysis coupled with the nonparametric nature of this method also prevented us from having sufficient sample size to split the sample to conduct validation analysis. Validation of the subgroups identified in this “first generation” study is recommended in subsequent investigations. The investigation would also have benefited from the addition of objective measures of the built environment that, while not necessarily more strongly related to obesity and other health outcomes than perceived measures [63], would have nonetheless provided an additional rich source of environmental information [64]. Unfortunately, such data collection was beyond the capabilities of the LIFE Trial. Similarly, the specific inclusion/exclusion criteria applied in the LIFE trial aimed at insufficiently active older adults at increased risk for mobility disability limits the potential generalizability of the results to healthier, more active groups of older adults. Finally, the relatively small number of African Americans/Blacks in this sample diminished statistical power with respect to potential further partitioning of the African American/Black subgroup. The relatively small number of participants contributed by each field center (~200 each) also diminished power with respect to achieving a thorough understanding of regional associations among the variables under study.

In conclusion, the current results suggest that, in combination with race/ethnicity, features of the perceived neighborhood built and social environments (specifically, perceived residential density and homophily) identified distinct subgroups of vulnerable older adults that differed in obesity prevalence. Pending additional verification, the results may help to inform the subsequent targeting of such subgroups for further investigation.

## Appendix

**Research Investigators for the LIFE Study**

The Lifestyle Interventions and Independence for Elders Study is funded by a National Institutes of Health/National Institute on Aging Cooperative Agreement

#UO1 AG22376 and a supplement from the National Heart, Lung and Blood Institute 3U01AG022376-05A2S, and sponsored in part by the Intramural Research Program, National Institute on Aging, NIH.

**Administrative Coordinating Center, University of Florida, Gainesville, FL**

Marco Pahor, MD – Principal Investigator of the LIFE Study

Jack M. Guralnik, MD, PhD – Co- Investigator of the LIFE Study (University of Maryland School of Medicine, Baltimore, MD)

Stephen D. Anton, PhD

Thomas W. Buford, PhD

Christiaan Leeuwenburgh, PhD

Susan G. Nayfield, MD, MSc

Todd M. Manini, PhD

Connie Caudle

Lauren Crump, MPH

Latonia Holmes

Jocelyn Lee, PhD

Ching-ju Lu, MPH

This research is partially supported by the University of Florida Claude D. Pepper Older Americans Independence Center (1 P30 AG028740).

**Data Management**, **Analysis and Quality Control Center**, **Wake Forest University**, **Winston Salem**, **NC**

Michael E. Miller, Ph.D. – DMAQC Principal Investigator

Mark A. Espeland, Ph.D. – DMAQC Co- Investigator

Walter T. Ambrosius, PhD

William Applegate, MD

Daniel P. Beavers, PhD, MS

Robert P. Byington, PhD, MPH, FAHA

Delilah Cook, CCRP

Curt D. Furberg, MD, PhD

Lea N. Harvin, BS

Leora Henkin, MPH, Med

John Hepler, MA

Fang-Chi Hsu, PhD

Kathy Joyce

Laura Lovato, MS

Juan Pierce, AB

Wesley Roberson, BSBA

Julia Robertson, BS

Julia Rushing, BSPH, MStat

Scott Rushing, BS

Cynthia L. Stowe, MPM

Michael P. Walkup, MS

Don Hire, BS

W. Jack Rejeski, PhD

Jeffrey A. Katula, PhD, MA

Peter H. Brubaker, PhD

Shannon L. Mihalko, PhD

Janine M. Jennings, PhD

**National Institutes of Health**, **Bethesda**, **MD**

Evan C. Hadley, MD (National Institute on Aging)

Sergei Romashkan, MD, PhD (National Institute on Aging)

Kushang V. Patel, PhD (National Institute on Aging)

**National Heart**, **Lung and Blood Institute**, **Bethesda**, **MD**

Denise Bonds, MD, MPH

**Field Centers**

**Northwestern University**, **Chicago**, **IL**

Mary M. McDermott, MD – Field Center Principal Investigator

Bonnie Spring, PhD – Field Center Co-Investigator

Joshua Hauser, MD – Field Center Co-Investigator

Diana Kerwin, MD – Field Center Co-Investigator

Kathryn Domanchuk, BS

Rex Graff, MS

Alvito Rego, MA

**Pennington Biomedical Research Center**, **Baton Rouge**, **LA**

Timothy S. Church, MD, PhD, MPH – Field Center Principal Investigator

Steven N. Blair, PED (University of South Carolina)

Valerie H. Myers, PhD

Ron Monce, PA-C

Nathan E. Britt, NP

Melissa Nauta Harris, BS

Ami Parks McGucken, MPA, BS

Ruben Rodarte, MBA, MS, BS

Heidi K. Millet, MPA, BS

Catrine Tudor-Locke, PhD, FACSM

Ben P. Butitta, BS

Sheletta G. Donatto, MS, RD, LDN, CDE

Shannon H. Cocreham, BS

**Stanford University**, **Palo Alto**, **CA**

Abby C. King, Ph.D. – Field Center Principal Investigator

Cynthia M. Castro, PhD

William L. Haskell, PhD

Randall S. Stafford, MD, PhD

Leslie A. Pruitt, PhD

Veronica Yank, MD

Kathy Berra, MSN, NP-C, FAAN

Carol Bell, NP

Rosita M. Thiessen

Kate P. Youngman, MA

Selene B. Virgen, BAS

Eric Maldonado, BA

Kristina N. Tarin, MS, CSCS

Heather Klaftenegger, BS

Carolyn A. Prosak, RD

Ines Campero, BA

Dulce M. Garcia, BS

José Soto, BA

Linda Chio, BA

David Hoskins, MS

**Tufts University**, **Boston**, **MA**

Roger A. Fielding, PhD – Field Center Principal Investigator

Miriam E. Nelson, PhD

Sara C. Folta, PhD

Edward M. Phillips, MD

Christine K. Liu, MD

Erica C. McDavitt, MS

Kieran F. Reid, PhD, MPH

Dylan R. Kirn, BS

Evan P. Pasha, BS

Won S. Kim, BS

Julie M. Krol, MS

Vince E. Beard, BS

Eleni X. Tsiroyannis, BS

Cynthia Hau, BS, MPH

Dr. Fielding's contribution is partially supported by the U.S. Department of Agriculture, under Agreement No. 58-1950-0-014. Any opinions, findings, conclusion, or recommendations expressed in this publication are those of the author(s) and do not necessarily reflect the view of the U.S. Dept of Agriculture.

This research is also supported by the Boston Claude D. Pepper Older Americans Independence Center (1P30AG031679) and the Boston Rehabilitation Outcomes Center (1R24HD065688-01A1).

**University of Florida**, **Gainesville**, **FL**

Todd M. Manini, PhD – Field Center Principal Investigator

Marco Pahor, MD – Field Center Co- Investigator

Stephen D. Anton, PhD

Thomas W. Buford, PhD

Michael Marsiske, PhD

Susan G. Nayfield, MD, MSc

Bhanuprasad D. Sandesara, MD

Mieniecia L. Black, MS

William L. Burk, MS

Brian M. Hoover, BS

Jeffrey D. Knaggs, BS

William C. Marena, MT, CCRC

Irina Korytov, MD

Stephanie D. Curtis, BS

Megan S. Lorow, BS

Chaitalee S. Goswami

Melissa A. Lewis

Michelle Kamen BS

Jill N. Bitz

Brian K. Stanton, BS

Tamika T. Hicks, BS

Charles W. Gay, DC

Chonglun Xie, MD (Brooks Rehabilitation Clinical Research Center, Jacksonville, FL)

Holly L. Morris, MSN, RN, CCRC (Brooks Rehabilitation Clinical Research Center, Jacksonville, FL)

Floris F. Singletary, MS, CCC-SLP (Brooks Rehabilitation Clinical Research Center, Jacksonville, FL)

Jackie Causer, BSH, RN (Brooks Rehabilitation Clinical Research Center, Jacksonville, FL)

Susan Yonce, ARNP (Brooks Rehabilitation Clinical Research Center, Jacksonville, FL)

Katie A. Radcliff, M.A. (Brooks Rehabilitation Clinical Research Center, Jacksonville, FL)

Mallorey Picone Smith, B.S. (Brooks Rehabilitation Clinical Research Center, Jacksonville, FL)

Jennifer S. Scott, B.S. (Brooks Rehabilitation Clinical Research Center, Jacksonville, FL)

Melissa M. Rodriguez B.S. (Brooks Rehabilitation Clinical Research Center, Jacksonville, FL)

Margo S. Fitch, P.T. (Brooks Rehabilitation Clinical Research Center, Jacksonville, FL)

Mendy C. Dunn, BSN (Assessment) (Brooks Rehabilitation Clinical Research Center, Jacksonville, FL)

Jessica Q. Schllesinger, B.S. Brooks Rehabilitation Clinical Research Center, Jacksonville, FL)

This research is partially supported by the University of Florida Claude D. Pepper Older Americans Independence Center (1 P30 AG028740).

**University of Pittsburgh**, **Pittsburgh**, **PA**

Anne B. Newman, MD, MPH – Field Center Principal Investigator

Stephanie A. Studenski, MD, MPH – Field Center Co-Investigator

Bret H. Goodpaster, PhD

Oscar Lopez, MD

Nancy W. Glynn, PhD

Neelesh K. Nadkarni, MD, PhD

Diane G. Ives, MPH

Mark A. Newman, PhD

George Grove, MS

Kathy Williams, RN, BSEd, MHSA

Janet T. Bonk, MPH, RN

Jennifer Rush, MPH

Piera Kost, BA (deceased)

Pamela Vincent, CMA

Allison Gerger, BS

Jamie R. Romeo, BS

Lauren C. Monheim, BS

The Pittsburgh Field Center is partially supported by the Pittsburgh Claude D. Pepper Older Americans Independence Center (P30 AG024827).

**Wake Forest University**, **Winston Salem**, **NC**

Stephen B. Kritchevsky, Ph.D. – Field Center Principal Investigator

Anthony P. Marsh, PhD – Field Center Co-Principal Investigator

Tina E. Brinkley, PhD

Jamehl S. Demons, MD

Kaycee M. Sink, MD, MAS

Kimberly Kennedy, BA, CCRC

Rachel Shertzer-Skinner, MA, CCRC

Abbie Wrights, MS

Rose Fries, RN, CCRC

Deborah Barr, MA, RHEd, CHES

The Wake Forest University Field Center is, in part, supported by the Claude D. Pepper Older Americans Independence Center (1 P30 AG21332).

**Yale University**, **New Haven**, **CT**

Thomas M. Gill, M.D. – Field Center Principal Investigator

Robert S. Axtell, PhD, FACSM – Field Center Co-Principal Investigator (Southern Connecticut State University, Exercise Science Department)

Susan S. Kashaf, MD, M.P.H. (VA Connecticut Healthcare System)

Nathalie de Rekeneire, MD, MS

Joanne M. McGloin, MDiv, MS, MBA

Raeleen Mautner, PhD

Sharon M. Huie-White, MPH

Luann Bianco, BA

Janice Zocher

Karen C. Wu, RN

Denise M. Shepard, RN, MBA

Barbara Fennelly, MA, RN

Rina Castro, LPN

Sean Halpin, MA

Matthew Brennan, MA

Theresa Barnett, MS, APRN

Lynne P. Iannone, MS, CCRP

Maria A. Zenoni, M.S.

Julie A. Bugaj, MS

Christine Bailey, MA

Peter Charpentier, MPH

Geraldine Hawthorne-Jones

Bridget Mignosa

Lynn Lewis

Dr. Gill is the recipient of an Academic Leadership Award (K07AG3587) from the National Institute on Aging.

The Yale Field Center is partially supported by the Claude D. Pepper Older Americans Independence Center (P30AG021342).

**Cognition Coordinating Center**, **Wake Forest University**, **Winston Salem**, **NC**

Jeff Williamson, MD, MHS – Center Principal Investigator

Kaycee M Sink, MD, MAS – Center Co-Principal Investigator

Hugh C. Hendrie, MB, ChB, DSc (Indiana University)

Stephen R. Rapp, PhD

Joe Verghese, MB, BS (Albert Einstein College of Medicine of Yeshiva University)

Nancy Woolard

Mark Espeland, PhD

Janine Jennings, PhD

Valerie K. Wilson, MD

**Electrocardiogram Reading Center**, **University of Florida**, **Gainesville**, **FL**

Carl J. Pepine MD, MACC

Mario Ariet, PhD

Eileen Handberg, PhD, ARNP

Daniel Deluca, BS

James Hill, MD, MS, FACC

Anita Szady, MD

**Spirometry Reading Center**, **Yale University**, **New Haven**, **CT**

Geoffrey L. Chupp, MD

Gail M. Flynn, RCP, CRFT

Thomas M. Gill, MD

John L. Hankinson, PhD (Hankinson Consulting, Inc.)

Carlos A. Vaz Fragoso, MD

Dr. Fragoso is the recipient of a Career Development Award from the Department of Veterans Affairs.

**Cost Effectiveness Analysis Center**

Erik J. Groessl, PhD (University of California, San Diego and VA San Diego Healthcare System)

Robert M. Kaplan, PhD (Office of Behavioral and Social Sciences Research, National Institutes of Health)
